# Transcription and Chromatin Organization of a Housekeeping Gene Cluster Containing an Integrated β-Globin Locus Control Region

**DOI:** 10.1371/journal.pgen.1000016

**Published:** 2008-03-07

**Authors:** Daan Noordermeer, Miguel R. Branco, Erik Splinter, Petra Klous, Wilfred van IJcken, Sigrid Swagemakers, Manousos Koutsourakis, Peter van der Spek, Ana Pombo, Wouter de Laat

**Affiliations:** 1 Department of Cell Biology and Genetics, Erasmus Medical Center, Rotterdam, The Netherlands; 2 MRC Clinical Sciences Centre, Faculty of Medicine, Imperial College London, London, United Kingdom; 3 Erasmus Center for Biomics, Erasmus Medical Center, Rotterdam, The Netherlands; 4 Erasmus Center for Bioinformatics, Erasmus Medical Center, Rotterdam, The Netherlands; Massachusetts General Hospital, United States of America

## Abstract

The activity of locus control regions (LCR) has been correlated with chromatin decondensation, spreading of active chromatin marks, locus repositioning away from its chromosome territory (CT), increased association with transcription factories, and long-range interactions via chromatin looping. To investigate the relative importance of these events in the regulation of gene expression, we targeted the human β-globin LCR in two opposite orientations to a gene-dense region in the mouse genome containing mostly housekeeping genes. We found that each oppositely oriented LCR influenced gene expression on both sides of the integration site and over a maximum distance of 150 kilobases. A subset of genes was transcriptionally enhanced, some of which in an LCR orientation-dependent manner. The locus resides mostly at the edge of its CT and integration of the LCR in either orientation caused a more frequent positioning of the locus away from its CT. Locus association with transcription factories increased moderately, both for loci at the edge and outside of the CT. These results show that nuclear repositioning is not sufficient to increase transcription of any given gene in this region. We identified long-range interactions between the LCR and two upregulated genes and propose that LCR-gene contacts via chromatin looping determine which genes are transcriptionally enhanced.

**Author summary**

Regulatory DNA elements are stretches of DNA that control transcription of genes even when they are located at a distance on the same chromosome. They are often included in DNA vectors to ensure transcription of rescue genes in gene therapy approaches. It is known that upon integration in the host genome, they can influence expression of host genes surrounding the integration site, but it is not known how they do so and over what distance they can function. Here, we targeted a prototype strong regulatory DNA element, the β-globin Locus Control Region, to a defined position in the mouse genome and we analyzed its effect on surrounding genes. We show that the locus control region can enhance transcription of at least seven genes that may be as far as 150 kilobases away. In the presence of the locus control region, the genes occupy a different location in the nucleus in a subset of cells, which may explain how some, but not all of these genes are activated. The locus control region also physically contacts the genes it activates by formation of DNA loops. We propose that these contacts are most important for the long-range activation of gene expression by regulatory DNA elements.

## Introduction

One of the main aims in the post-genomic era has been to understand how genes are regulated at the level of transcription, giving rise to cell-type specific transcriptomes. Most of our knowledge on the regulation of gene transcription is biased towards studies of a small number of atypical genes showing highly restricted expression patterns. Expression of these tissue-specific genes is often controlled by distant transcription regulatory DNA elements. The β-globin locus control region (LCR) is a prototype of a strong mammalian regulatory DNA element. At its endogenous position, the LCR enhances the expression of the mouse β-globin-like genes 25–100 fold [1]. LCR-mediated transcriptional enhancement has been correlated with chromatin opening [2], histone acetylation of the locus (in case of the LCR of the human growth hormone cluster) [3], the initiation of intergenic transcripts [4],[5], the spreading of a histone methyltransferase [6] and intrachromosomal interactions with active genes via chromatin looping [7],[8]. At the level of nuclear organization, LCR activity has been correlated with repositioning of loci away from their chromosome territory (CT) and towards the nuclear interior, and increased association with transcription factories [9],[10]. However, the relative importance of these events for the enhancement of gene transcription is currently unclear.

Remarkably little is known about the regulation of more ubiquitously expressed genes, which comprise the major part of the coding genome. High-throughput expression studies revealed that housekeeping genes often cluster in large gene-dense regions on mouse and human chromosomes [11],[12]. Breakpoints of synteny were shown to be under-represented in these regions, suggesting that this organization is under natural selection [13]. Gene expression seems to benefit from clustering along the linear genomic sequence. When genes are integrated at random positions in the genome, their expression is often subject to position effect variegation [2]. Furthermore, reporter genes express at higher levels when integrated in active gene-dense regions, indicating the existence of domain-wide regulatory mechanisms [14].

It has been suggested that gene clustering promotes the maintenance of transcriptionally competent domains of open or decondensed chromatin across the gene-dense region, with expression of individual genes being dictated by the availability of specific transcription factors [14],[15]. Another emerging hypothesis is that clustered genes may collectively stabilize their position at nuclear zones of increased transcriptional competence, which in turn would positively affect the expression levels of genes within the cluster [12],[16]. The location of genes relative to several nuclear landmarks has been correlated with gene expression. For example, activation of MHCII cluster genes, epidermal differentiation complex (EDC) and Hox genes promotes a large-scale relocation of the subchromosomal regions that contain them, away from the respective CT [17],[18],[19]. In general however, there seems to exist little correlation between gene activity and position versus the CT [20],[21],[22],[23],[24].

Proximity of genomic regions to nuclear structures rich in the RNA processing machinery has also been found to correlate with increased expression. Gene-rich R-bands are more frequently associated with ‘splicing speckles’ than their gene-poor counterparts, G-bands [25], and transcriptional activity of the globin genes also correlates with increased association with speckles [26]. Whilst it is clear that transcription, and therefore co-transcriptional splicing, can occur both away and near splicing speckles, it appears that locus association with these structures may facilitate mRNA processing, especially in the case of intron-rich transcripts, such as COL1A1 [27]. Lastly, proximity to clusters of multiple active RNA polymerases (RNAP), known as transcription factories, may facilitate expression of genes that lie adjacent on the linear DNA template [28],[29],[30],[31]. Live-cell imaging of GFP-tagged RNAP II shows that RNAP II complexes are extremely mobile and can access the whole nucleoplasm [32],[33]. When active, RNAP II is immobile and found in transcription factories that contain on average 8 active RNAP II complexes [29],[34],[35]. Irrespective of cell type, the size of factories as determined by electron microscopy is 50–70 nm [29],[30],[34]. Differences in transcriptional activity amongst different cell types correlate with the number of factories present per nucleus, rather than with changes in factory size [30].

While it is clear that gene expression is controlled at various levels, it is difficult to assess the hierarchy and importance of each level of regulation, as many of the observations are made on different gene loci and in different types of cells, often cultured in vitro. Here, we generated transgenic mice, isolated primary tissues and analyzed in detail gene expression, chromatin structure and nuclear positioning of a gene-dense region in the absence or presence of an integrated β-globin LCR in opposite orientations. We found that insertion of the LCR induced a relocation of the locus away from the edge of its CT and increased the association with RNAP II factories. The latter occurred independently of the locus being positioned at the edge or outside the CT. Changes in gene expression occurred bi-directionally and as far as 150 kb from the site of LCR insertion, but were variable for individual genes, depending on the orientation of the integrated LCR. The fact that both orientations of the LCR caused a repositioning of the locus away from its CT, yet genes within the locus responded selectively to only one LCR orientation, shows that the observed repositioning is not the only factor determining the increase in transcription of the individual genes. The gene expression patterns seem incompatible with tracking or spreading mechanisms, and we demonstrate that the LCR physically interacts with the two most upregulated genes. We propose that LCR-gene contacts via chromatin looping are key to the upregulation of at least a number of genes in this gene-dense region.

## Results

### Introduction of the β-globin LCR into a gene-dense region

**Figure 1:**
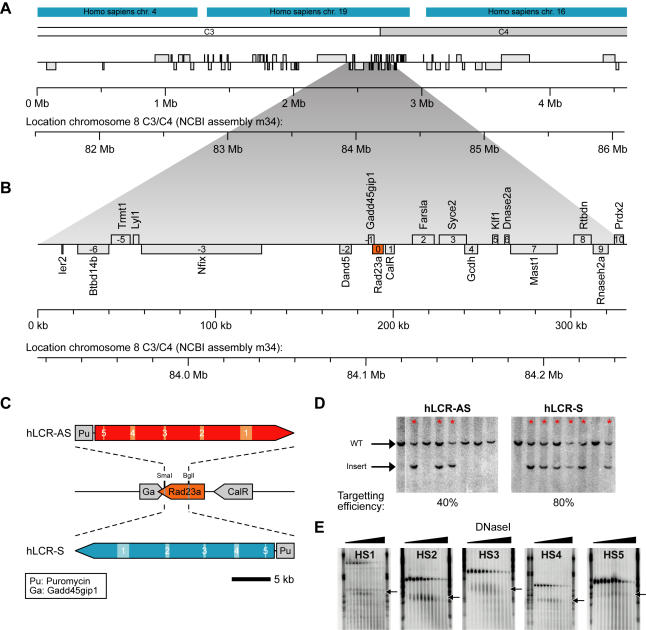
Targeting of the β-globin LCR into the murine gene-dense region 8C3/C4. (A) 3 Mb region containing 79 genes (NCBI assembly m34). Location on murine chromosome 8 is depicted below the region. Human synteny and cytogenic chromosome bands are depicted on top. (B) Zoom in on 300 kb region containing 18 genes characterized in this study (NCBI assembly m34). Genes are numbered relative to the center gene *Rad23a* (gene 0). Location on murine chromosome 8 is depicted below the region. (C) The full human β-globin LCR, linked to a puromycin selection marker, was integrated in sense orientation (LCR-S, blue) and anti-sense orientation (LCR-AS, red) relative to *Rad23a*, removing exons II–VII from the *Rad23a* gene. Hypersensitive sites (HSs) in the LCR are numbered and represented by shaded boxes. (D) Southern blot showing targeting of the LCR into the *Rad23a* locus. Positive clones are indicated by red arrows. Note that targeting is extremely efficient. (E) DnaseI hypersensitivity of HSs in the integrated LCR in the sense orientation. E14.5 fetal liver DNA was digested with increasing amounts of DnaseI and DNA fragments containing the respective HSs were visualized by Southern blotting. Hypersensitive DNA fragments are indicated by arrows. LCR-AS HSs showed comparable patterns (not shown).

In order to study the relationship between chromatin structure, nuclear organization and transcription regulation, we introduced the human β-globin LCR in two orientations into a region on mouse chromosome 8 that contains 18 genes within 300 kb. This extremely gene-dense region, which we refer to as 8C3/C4, is located within a 3 Mb gene cluster with 79 genes that resides in the middle of chromosome 8 at the transition of band C3 and C4 (NCBI assembly m34) (Figure 1A and 1B). The genes at 8C3/C4 do not share sequence homology and encode for proteins that function in very diverse cellular processes, suggesting that gene clustering at this genomic location is not the result of sequence duplication during evolution.

The β-globin LCR is a strong, erythroid-specific, regulatory DNA element that confers position-independent and copy-number dependent expression to transgenes in mice [2]. We targeted the full, ∼21 kb, human LCR in sense (S) and anti-sense (AS) orientations into the *Rad23a* gene (gene 0 in Figure 1B) using homologous recombination in ES cells (Figure 1C and 1D). *Rad23a* is located centrally in 8C3/C4 and can be knocked out on both alleles without getting an apparent change in phenotype, due to the redundant presence of the homologous *Rad23b* gene in the mouse genome [36]. Mice homozygous for the integrated LCR in both orientations also did not show an abnormal phenotype. To test for functionality of the integrated LCR, DNaseI hypersensitivity assays were performed. Each of the 5 hypersensitive sites (HSs) characteristic for the human β-globin LCR were found to be present in E14.5 liver cells of LCR-S +/+ and LCR-AS +/+ fetuses, indicating that the integrated LCR binds its normal repertoire of transcription factors (Figure 1E).

### Gene expression at 8C3/C4 in the presence of an integrated LCR

**Figure 2:**
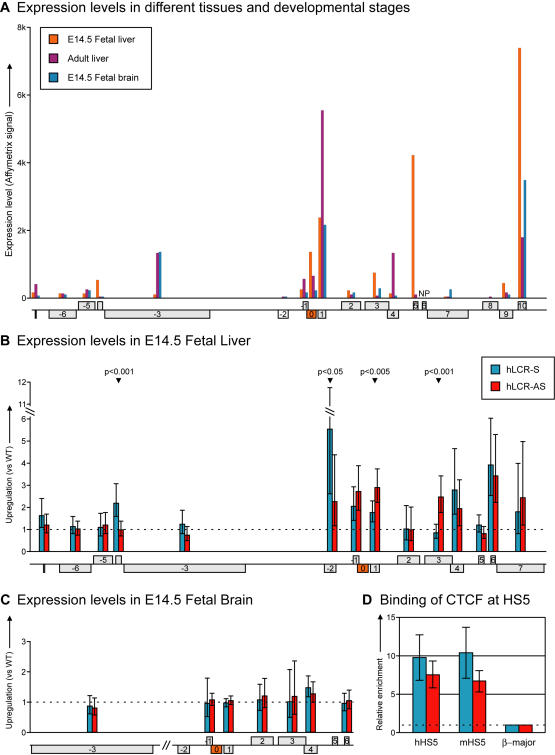
Gene expression in different tissues at 8C3/C4. (A) Expression levels in fetal liver, fetal brain and adult liver of genes at 8C3/C4 represented on murine Affymetrix 430 2.0 Micro-array. NP: not present on the micro-array. (B) Upregulation of gene expression relative to WT levels (for each gene set at 1) in strains with the human β-globin LCR integrated at the *Rad23a* locus (orange). Expression levels were determined in E14.5 fetal liver using qRT-PCR. Error bars depict 95% confidence intervals obtained from a Student's t-test using the Welch-Satterthwaite approximation for the degrees of freedom. P-values of significant differences in expression levels measured between the two oppositely LCRs are shown above the relevant genes. (C) Upregulation of gene expression relative to WT levels (for each gene set at 1) in E14.5 fetal brain containing the human β-globin LCR. Expression levels were determined using qRT-PCR. Error bars depict 95% confidence intervals. (D) Binding of CTCF at HS5 of the human β-globin LCR (hHS5), as determined by ChIP-analysis in E14.5 fetal liver. Enrichments were normalized to the endogenous β-major promoter. Endogenous β-globin HS5 (mHS5) is shown as a control. Error bars represent SE of at least two independent experiments.

To gain insight into the transcriptional organization of the wild-type locus, we first performed a series of Affymetrix expression array experiments in various tissues (Figure 2A). We found that most of the genes within the region were expressed in all tissues, but that within a given tissue expression levels varied greatly between the genes. A few genes expressed only in a certain cell type whereas others showed considerable differences in transcription levels between tissues, which we found to reflect differences in transcription rates (Figure S1). This apparent lack of co-regulation between the closely juxtaposed genes is in agreement with genome-wide analyses showing that expression of individual genes does not correlate over distances of more than two genes [37].

We then studied the effect of the integration of the LCR in either orientation by quantitative reverse transcriptase PCR (qRT-PCR). A complex pattern of transcriptional upregulation around the integration site was observed in E14.5 livers (Figure 2B). To exclude that integration *per se*, or the disruption of the *Rad23a* gene, had an effect on gene expression at 8C3/C4, we also analyzed transcription of several genes in E14.5 brain cells, which do not contain LCR activity. No upregulation of genes in the region was observed in these cells (Figure 2C), with the exception of one gene, which appears ∼1.5 fold upregulated in one of the transgenic lines. Possibly, this is due to its altered genetic background. We conclude that the complex pattern of transcriptional upregulation in fetal liver cells is dependent on the erythroid-specific LCR activity.

In fetal liver, each oppositely oriented LCR enhanced the expression of at least seven genes surrounding the integration site, the most distal one (*Ier2*, gene −7) being over 150 kb away from the integrated LCR. Both LCRs activated genes on the plus and minus strands, as well as upstream and downstream of the integration site, demonstrating bi-directional activity of the LCR. The latter observation was somewhat surprising given that the LCR is thought to function in a unidirectional manner in the β-globin locus [38]. However, this finding was in agreement with results showing that a marked β-globin gene placed upstream of the LCR competes with downstream genes for activation by the LCR [39]. We found that upstream gene activation occurred despite the binding of the insulator protein CTCF to the outer hypersensitive site (HS5) of the LCR (Figure 2D), confirming that binding of CTCF does not necessarily lead to enhancer-blocking [40].

Genes present in 8C3/C4 reacted very differently to the integration of the two LCRs (Figure 2B). Genes at positions −1 (*Gadd45gip1*), +4 (*Gcdh*), and +6 (*Dnase2a*) relative to the integration site (defined as gene position 0) were upregulated to levels that were similar between both LCRs. Genes at positions −2 (*Dand5*) and +1 (*CalR*) also responded to both LCRs, but reached significantly different levels of mRNA. Interspersed, at positions −6 (*Btbd14b*), −5 (*Trmt1*), −3 (*Nfix*), +2 (*Farsla*) and +5 (*Klf1*) were genes not responding to the LCR in either orientation. We reasoned that structural constraints or a lack of certain *cis*-regulatory elements prevented these genes from communicating with the LCR, or that they were transcribed already at maximum rates in wild-type cells. The finding that gene +5 (*Klf1*) is not upregulated by the LCR is particularly interesting, since this is the only gene in the region that is specifically expressed in erythroid cells. This suggests that upregulation of genes by the LCR is not determined by a shared functional relationship. Finally, the gene at position −4 (*Lyl1*) was significantly upregulated only by the LCR integrated in the sense (S) orientation, whereas the gene at position +3 (*Syce2*) responded significantly only to LCR-AS.

### Nuclear repositioning of 8C3/C4 by the integrated LCR

**Figure 3:**
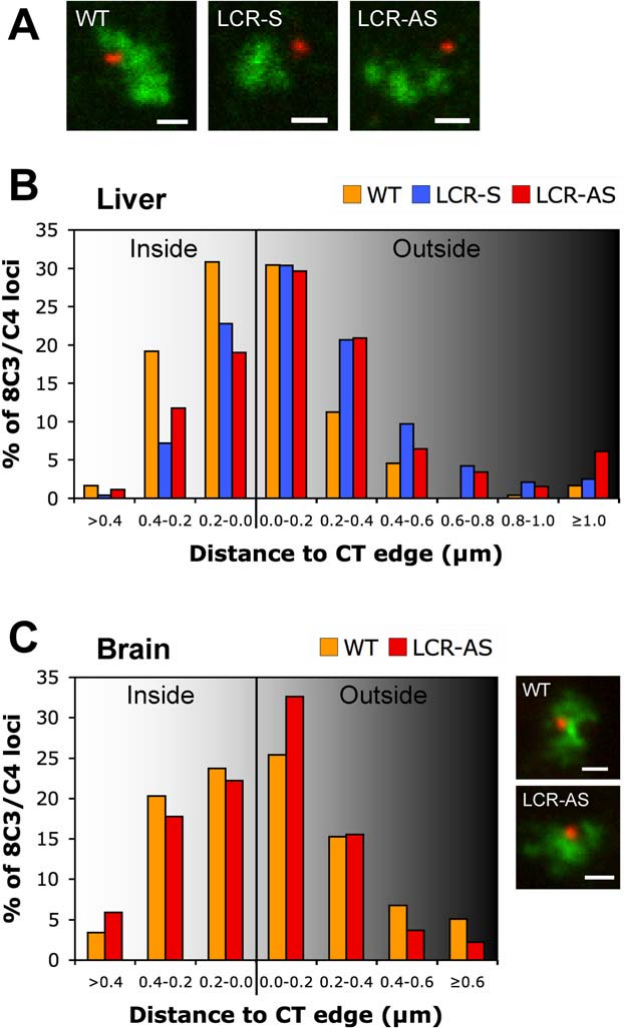
Effect of LCR insertion on the positioning of 8C3/C4 in relation to its CT. (A) Cryosections from WT, LCR-S and LCR-AS fetal liver nuclei were hybridized to a BAC probe containing the 8C3/C4 locus (red) and a chromosome 8 specific paint (green). Bars, 1 µm. (B) After cryo-FISH, the distances from the center of each locus to the nearest CT edge were measured (n≥237 loci). Statistically significant differences were found between the wild-type locus and both transgenic loci containing the LCR (p<0.001, K-S test). (C) Distances between 8C3/C4 and the CT edge were measured for sections from WT (n = 59 loci) and LCR-AS (n = 135 loci) fetal brain, showing no differences between the two populations (p = 0.45, K-S test). Bars, 1 µm.

The LCR has been implicated in nuclear repositioning of loci and we therefore analyzed whether LCR insertion had an effect on the position of 8C3/C4 relative to the CT, the nuclear periphery, transcription factories and splicing speckles. We first analyzed the position of 8C3/C4 with and without the LCR (using a ∼170 kb BAC probe) relative to its own CT (labeled with a whole chromosome 8 probe) in E14.5 fetal liver and brain cells (Figure 3). For this, we performed cryo-FISH (fluorescence in situ hybridization on thin cryosections), a novel technology that offers increased resolution in the z-axis (150–200 nm) compared to standard 2D- and 3D-FISH protocols (>500 nm) [24]. In the LCR samples, the BAC signal had a similar dot-like appearance to wild-type cells, despite the insertion of the 21 kb transgenic fragment (Figure 3A). The distances between the center of each locus and the nearest CT edge were then measured. As observed previously for other actively transcribed gene clusters [23], WT 8C3/C4 in fetal liver was already found mostly near the edge of its CT (61% within 0.2 µm of the edge), and 7% was >0.4 µm away from the edge, looping out from the CT (Figure 3B). Insertion of the LCR in either orientation caused a highly significant shift of the distribution of distances between 8C3/C4 and the CT edge towards a more external position (p<0.001; K-S test). The percentage of loci found looped out at >0.4 µm away from the CT increased from 7% in wild-type to 19% in LCR-S and 17% in LCR-AS mice (Figure 3B). Thus, the LCR induces relocation of 8C3/C4 away from its CT, independently of its orientation in the locus. In wild-type brain cells, 8C3/C4 is also found at the periphery of its CT but does not relocate after LCR insertion (Figure 3C). As the LCR is inactive in brain cells, these results suggest that the repositioning of the locus in liver cells is dependent on LCR activity. The repositioning of 8C3/C4 relative to its CT does not reflect a change in radial nuclear position, as both wild-type and LCR-integrated loci occupy the same preferred radial position away from the nuclear periphery in fetal liver (data not shown).

We next performed a cryo-immuno-FISH experiment to determine the association of 8C3/C4 with splicing speckles, which are the nucleoplasmic regions enriched for splicing factors [41]. We found the WT region often adjacent to (63%) or overlapping with (21%) splicing speckles labeled with the Sm antigen, a spliceosomal factor (Figure S2), as would be expected for a region containing many active genes [25]. Integration of the LCR had no effect on interaction frequencies with nuclear speckles, which may be due to the already high interaction frequency of the WT locus. As splicing speckles are located outside CTs, the preferred association of 8C3/C4 with these domains is consistent with the favored position of the locus towards the periphery or outside of its CT.

**Figure 4:**
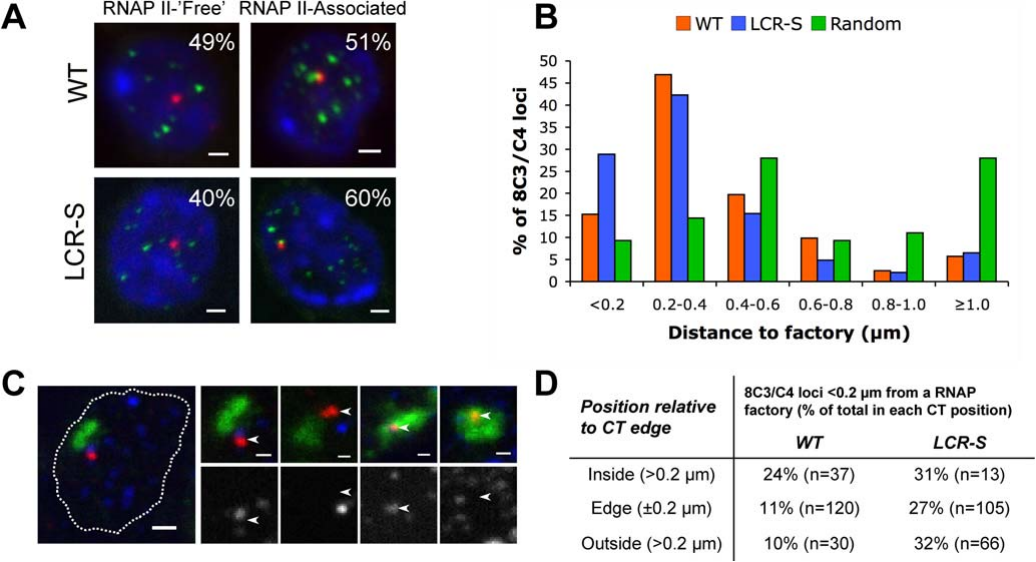
Effect of LCR insertion on the association of 8C3/C4 with transcription factories. (A) Nuclear sections from WT and LCR-S fetal livers (blue) were immunolabeled with an antibody against the Ser2-phosphorylated form of RNAP II (green), hybridized to the 8C3/C4 BAC probe (red), and the association of the locus with transciption factories was scored (n≥231 loci). Bars, 1 µm. (B) To obtain an unbiased measurement of factory association, distances were measured between the centres of 8C3/C4 loci and the nearest RNAP II focus, showing significant differences in the distances distributions between WT and LCR-S loci (p = 0.003, K-S test). Both loci are found in closer proximity to transcription factories than predicted by a random model (p<0.001, K-S test), in which loci were randomly placed inside experimental images of RNAP II-labeled nuclei and distances measured. (C) The active form of RNAP II (blue or grayscale), 8C3/C4 (red; arrows) and chromosome 8 (green) were labeled as before. The nucleus is outlined by a white line. Bars, 1 µm. (D) Distances of 8C3/C4 loci to the nearest CT edge and transcription factory were measured after performing the triple labeling described above (C) in sections from WT and LCR-S fetal livers. The frequency of loci <0.2 µm away from a transcription factory was calculated within each position relative to the CT.

We then analyzed whether the LCR influenced the association of 8C3/C4 with foci containing the transcriptionally active (serine2-phosphorylated) form of RNAP II, which marks transcription factories as determined after co-localization with sites of Br-UTP incorporation [29],[42]. Using cryo-immuno-FISH to simultaneously visualize the elongating isoform of RNAP II and 8C3/C4, we found that 51% of the WT alleles associated with RNAP II foci, as scored visually, meaning that signals overlapped or touched (without background pixels in between; Figure 4A). Integration of the β-globin LCR had a small positive effect on the frequency of association of 8C3/C4 with RNAP II foci, increasing from 51% to 60% in wild-type versus LCR-S +/+ fetal liver sections (p = 0.03; Fisher's test using pooled data from two independent hybridizations). To more carefully determine the association of 8C3/C4 with factories in an unbiased fashion, we performed distance measurements between the centers of the fluorescent signals (Figure 4B). The two distance distributions differed significantly, with loci in LCR-S cells being closer to transcription factories than those in WT cells (p = 0.003, K-S test), confirming the previous results. The largest difference observed in individual distance categories was for loci closest to a factory (<0.2 µm). In wild-type cells 15% of the loci were scored in this category, while this percentage doubled to 29% in LCR-S cells. To test whether these frequencies may also be explained by random, non-functional associations with RNAP II foci, we performed an in silico experiment. We used the experimental images of nuclei labeled with RNAP II and applied a computational algorithm to generate a randomly positioned locus within each nuclear section. We subsequently measured the distance to the nearest factory and found that the overall distribution of randomly positioned loci was very different from the distributions measured for the wild-type and the LCR-containing locus (Figure 4B; p<0.001, K-S test), which were more frequently close to the factories. These results suggest that the measured associations between 8C3/C4 loci and transcription factories reflect functionally significant interactions that are increased in the presence of the LCR. The increased factory association may reflect the increased overall transcriptional activity of the region and/or be a consequence of the capacity of the LCR to recruit RNAP II.

Given the concomitant relocation of the locus away from the CT and its increased association with RNAP II in the presence of the LCR, we tested whether LCR-mediated looping out from the CT was driving the increased association with RNAP II foci. We simultaneously visualized 8C3/C4, the territory of chromosome 8 and the active form of RNAP II in cryosections (Figure 4C). Distances between 8C3/C4 and, respectively, the CT edge and RNAP II foci were measured, and RNAP II association frequencies (<0.2 µm) were scored for loci inside, outside and at the edge (±0.2 µm) of the CT (Figure 4D). The data showed that in the presence of the LCR, the association frequency of 8C3/C4 with RNAP II foci increases independent of its position relative to the CT (Figure 4D). Logistic regression analysis shows that the increased association of 8C3/C4 with RNAP II is not dependent on CT position (p = 0.26), but solely on the presence of the LCR (p = 0.0002). Logistic regression also indicates that the apparently smaller effect of the LCR insertion for loci inside the CT is not statistically different from effects at the edge or outside the CT, although we cannot exclude that a regional effect was not observed due to the small number of loci observed in this region. Additionally, we found no correlation between distance to the CT edge and distance to transcription factories for both WT (R = 0.01) and LCR-S (R = 0.08) cells. The data suggest that LCR-induced looping out of 8C3/C4 from the CT is not a prerequisite for more frequent association with RNAP II foci. This is in agreement with the observation that active transcription factories are present within CTs, thus transcribing loci that are positioned inside CTs [21],[22],[24],[43].

### Chromatin organization at 8C3/C4 in the presence of an integrated LCR

**Figure 5:**
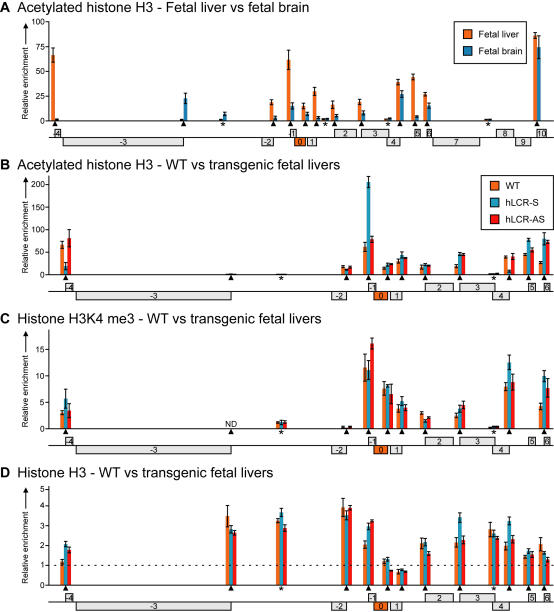
Chromatin organization at 8C3/C4 in the presence of the LCR. (A) Histone H3 acetylation at promoters (▴) and other regions (*) of 8C3/C4 in WT E14.5 fetal liver and brain as determined by ChIP-analysis. Enrichments were normalized to the Amylase promoter. (B) Histone H3 acetylation at 8C3/C4 in WT and transgenic strains in E14.5 fetal liver. Enrichments were normalized to the Amylase promoter. (C) Histone H3 lysine 4 tri-methylation at 8C3/C4 in WT and transgenic strains in E14.5 fetal liver. Enrichments were normalized to the endogenous β-major promoter. (D) Histone H3 occupancy at 8C3/C4 in WT and transgenic strains in E14.5 fetal liver. Enrichments were normalized to the endogenous β-major promoter. Error bars in all graphs represent standard error (SE) of at least two independent experiments.

We next investigated whether LCR-mediated upregulation of gene expression was caused by the spreading of an epigenetic signal from the LCR to the neighboring genes. In the human growth hormone cluster, the spreading of histone H3 acetylation from its LCR towards the target genes has been suggested to underlie the enhancement of their expression [3],[44] and to be responsible for the bystander activation of an unrelated gene present in the region [45], but the mechanism of such spreading is unknown. Recently it was also suggested that the β-globin LCR recruits an MLL2-containing protein complex, which after LCR-binding, would dissociate to allow for the spreading of the histone methyltransferase MLL2 and subsequent H3K4 methylation specifically at the active gene promoter [6]. Both acetylation of histone H3 and di- and trimethyl H3K4 are associated with active chromatin and on a genome-wide basis the levels of trimethyl H3K4 appear to correlate with transcriptional activity [46],[47]. We first performed chromatin immunoprecipitation (ChIP) experiments on WT E14.5 fetal liver and fetal brain to analyze the distribution of histone H3 acetylation (Figure 5A). A region-wide analysis in both tissues revealed enrichment of hyperacetylated histone H3 at the promoters of the actively transcribed genes, but not at the inactive promoters or intergenic areas present in the region (Figure 5A). Such punctuated, rather than domain-wide, pattern of hyperacetylation was previously observed at gene-rich regions in a genome-wide mapping study [48]. Tissue-specific genes such as *Nfix* (−3; active in fetal brain) and *Klf1* (+5; active in fetal liver) switch promoter acetylation status in relation to their altered expression status in the two tissues. We subsequently analyzed the effect of the LCR on histone acetylation levels at 8C3/C4. We found a marked change in the level of acetylation at a site immediately downstream of the integrated LCR-S, and no or minor changes at 12 other positions in the locus (Figure 5B). Similar observations were made when we analyzed H3K4 trimethylation across 8C3/C4, although here we noticed that increased levels of H3K4 tri-methylation corresponded with elevated expression levels at some genes (−4, +4, +6) (Figure 5C). In general though, the minor changes observed in the presence of the LCRs in acetylation and trimethylation levels of histone H3 appeared not to be strictly related to each other or to changes in transcriptional activity. Most likely, the changes in gene expression levels (2 to 6-fold) are too subtle to be reflected by changes in histone modification patterns. To analyze whether our data were influenced by differences in histone H3 occupancy, we performed ChIP with an antibody against the C-terminus of histone H3 against a subset of sites in WT and transgenic livers (Figure 5D). Considerable differences in H3 enrichment were observed in a pattern generally opposite to the one found with the antibodies against acetylated and trimethylated H3. Thus, H3 was most abundantly present at intergenic regions and at inactive promoters and relatively depleted from active promoters, as seen before [49]. Our data do not provide evidence for spreading of histone H3 acetylation or H3K4 trimethylation from the integrated LCRs. This is in agreement with the observation that deletion of the endogenous β-globin LCR has little effect on histone H3 acetylation patterns elsewhere in the β-globin locus [50]. We argue that the complex pattern of transcriptional up-regulation observed with the two LCRs is difficult to explain by a mechanism involving the linear spreading of a signal from the LCR.

**Figure 6:**
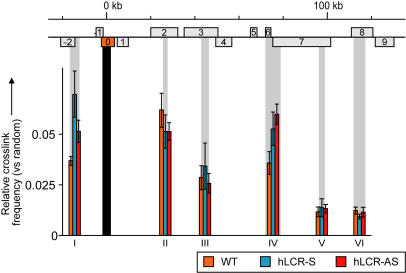
Chromatin looping at 8C3/C4 in the presence of the LCR. Relative crosslink frequencies (vs. random) in a part of the 300 kb region containing the two most upregulated genes (genes −2, *Dand5* and +6, *Dnase2a*). Crosslink frequencies between the fixed *BglII* fragment, depicted by a black bar, containing either the WT Rad23a gene or the LCR and other fragments, depicted by gray bars are visualized, as determined by 3C analysis and quantified by qPCR in WT and transgenic E14.5 fetal liver. Note that for the fixed fragment in each strain the same primer and Taqman probe combination was used. Error bars represent SE of at least three independent experiments.

Chromatin looping was previously observed at the human and mouse β-globin locus, where the LCR was found to specifically contact the actively transcribed β-globin genes [7],[8],[51]. To investigate whether increased expression levels were due to looping of the integrated LCR with specific genes at 8C3/C4, 3C technology (Chromosome Conformation Capture) was applied [52],[53], using a recently developed Taqman approach for a more accurate detection of crosslinked ligation products [54]. We focused on a *Rad23a* restriction fragment that spanned the integration site and designed one primer/probe combination that could be used for the analysis of interactions with the *Rad23a* gene in wild-type fetal liver cells, as well as for the analysis of interactions with the integrated LCR in transgenic cells (Figure 6). We found that upon introduction of the LCR, interaction frequencies increased specifically between this site and two fragments that contained the promoters of the two genes that were most activated by the integrated LCRs, suggesting that the LCR-dependent upregulation of these genes is mediated by chromatin looping (Figure 6). None of the other restriction fragments analyzed across 8C3/C4 showed a significant difference in interaction frequency between the wild-type and transgenic loci, not even when the fragments analyzed were at the promoters of up-regulated genes. This is not unexpected, as the LCR has been shown to contact only one gene at a time [55], such that increased contacts with the large number of target genes at 8C3/C4 will average out and be below the threshold of detection in the population of cells analyzed. In combination with previous observations made in the β-globin locus [7],[8],[51], we conclude that physical interactions between the LCR and at least the genes at +6 (*DNaseII*) and −2 (*Dand5*) are likely to be key to their transcriptional enhancement.

## Discussion

We have investigated the effect of the integration of the β-globin LCR into a gene-dense region containing many housekeeping genes. Two transgenic mouse lines were generated, each containing an oppositely oriented LCR inserted at the same genomic position. Gene activity, chromatin modifications and chromatin structure were analyzed across a large genomic region containing the integration site. In addition, detailed studies were performed on the position of the locus relative to its chromosome territory, the nuclear periphery and nuclear entities such as speckles and transcription factories. To our knowledge, this is the first study that analyzes in detail the impact of an integrated LCR on its surrounding genes and chromatin. As the wild-type region is already highly active, some of the changes induced by the LCR are subtle. More profound effects may be expected if the LCR is integrated at less active genomic positions. Nevertheless, our results demonstrate that the LCR can act on a relatively large number of genes (at least seven) spread over at least 150 kb, causes nuclear repositioning of the targeted locus and physically contacts the most upregulated genes via chromatin looping. Collectively, the data provide an integrated view on LCR functioning and on the various levels that control gene expression within a gene-dense region.

Gene activation has been correlated with repositioning of loci away from nuclear landmarks such as CTs, pericentromeric heterochromatin or the nuclear periphery. One interpretation of this phenomenon is that relocation critically drives transcription by positioning loci in nuclear zones of increased transcriptional competence. This idea may predict some degree of co-regulation between genes closely juxtaposed on the chromosome template [16]. We showed that introduction of the β-globin LCR into a gene-dense region of functionally unrelated genes caused (1) the more frequent positioning of this locus away from its CT, (2) a small but significant increase in association frequency with transcription factories, (3) no change in association frequency of the locus with splicing speckles and no change in the nuclear radial position. We also found an increase in expression levels of multiple genes surrounding the integration site. LCR-driven repositioning of the locus may facilitate increased transcriptional activity. Nevertheless, we consider it unlikely that the subset of cells in which 8C3/C4 is looped out from the CT can account for the overall increased levels of expression. Transcription also takes place at the interior of CTs [21],[22],[23],[24] and we show here that 8C3/C4 association with transcription factories increases both for loci at the edge and outside the CT. Moreover and as discussed below, our data demonstrate that at least for some genes repositioning is not sufficient to drive increased transcriptional activity.

Transcriptional enhancement was maximally 4–6 fold, which is modest compared to the impact the mouse LCR has at its endogenous position, where it increases β-major gene expression 25–100 fold [2]. An explanation for this difference could be that more genes compete for interaction with the LCR at 8C3/C4. Furthermore, gene promoter intrinsic properties could preclude that the LCR increases their expression more efficiently. In agreement with this notion is the fact that both the mouse and human β-globin locus contain a second adult β-globin-like gene (called β-minor and δ-globin, respectively) that is activated much less dramatically than their prototypic counterparts (β-major and β-globin). For example, mouse β-minor expression is 10-fold less than β-major expression and the difference between human δ- and β-globin gene expression is even more pronounced.

When we integrated an oppositely oriented LCR at the same genomic position at 8C3C4, it had an identical impact on the positioning of the locus relative to these nuclear landmarks, but upregulated a partially overlapping yet different set of genes. Previously it has been shown that tissue-specific genes surrounded by housekeeping genes maintain their inactive status upon nuclear repositioning in unrelated tissues [26]. This observation may be explained by the absence of cell-type specific transcription factors in these tissues [15]. In another recent study, *Lnp*, a gene located near the *Hoxd* cluster, was shown to be active and not change its expression level upon looping away from its CT during ES cell differentiation [43]. We observed a similar phenomenon at 8C3/C4, where genes at position −6 (*Btbd14b*), −5 (*Trmt1*), −3 (*Nfix*), +2 (*Farsla*) and +5 (*Klf1*) did not change their expression. An explanation for this may be that gene or chromatin intrinsic properties preclude the more efficient transcription of these genes. Importantly, the genes at position −4 (*Lyl1*) and +3 (*Syce2*), and to a less significant extent −7 (*Ier2*) increased their transcription activity, but responded to only one orientation of the LCR (as confirmed independently by Affymetrix microarray expression analysis (data not shown)). This shows that at least for these genes repositioning is not sufficient to drive their upregulation and that other mechanisms are involved.

Transcription regulation by the β-globin LCR has also been associated with the spreading of histone acetylation (in case of the LCR of the human growth hormone cluster) [3] and of the methyltransferase MLL2 [6]. We find the same punctuated pattern of acetylated and lysine-4 methylated histone H3 specifically at the active promoters of 8C3/C4 before and after LCR integration and find no evidence for spreading of these marks. The data do not exclude that the LCR attracts MLL2 and subsequently spreads the methyltransferase across the locus. However, the finding that genes distant to the LCR are upregulated, while genes more proximal are not, is difficult to explain by a mechanism involving the linear spreading of any signal from the LCR. If spreading occurs, it also not clear why a gene like *Syce2* (+3) is upregulated by the LCR in one, but not the other, orientation.

Previously, we have shown that the β-globin LCR contacts the active genes in the β-globin locus [8],[51]. Here, we find such contacts being formed between the LCR and the most strongly upregulated genes, suggesting that the ectopic LCR also acts by looping. Looping is thought to result from random collisions between chromatin sites that are stabilized when proteins bound to these sites have affinity for each other [56]. Alternatively, looping could also be the outcome of the LCR tracking along the intervening chromatin fiber towards gene promoters. The latter seems less compatible with our observation that LCR-S fails to activate *Syce2* at position +3 (a gene that is upregulated by LCR-AS), while being able to upregulate the genes at position +4 and +6 further down the chromatin fiber. Spatial proximity of the LCR with active genes is thought to increase the local concentration of transcription factors and/or RNA polymerase II, which might allow for more efficient transcription [56],[57]. Productive loop formation is predicted to depend on affinities between trans-acting factors bound to the LCR and to the gene. Thus, at 8C3/C4, the genes at position −6 (*Btbd14b*), −5 (*Trmt1*), −3 (*Nfix*), +2 (*Farsla*) and +5 (*Klf1*) that are not upregulated in the presence of an LCR would lack proteins that can interact with LCR-associated factors. This is especially surprising for gene +5 (*Klf1*), since it encodes an erythroid specific transcription factor, and therefore could be expected to bind similar trans-acting factors to the promoter as the LCR. We notice, however, that an erythroid-specific enhancer less than 1 kb upstream of the promoter acts on this gene and may compete out the LCR. Why would the productive formation of a loop be dependent on the orientation of the LCR, as was found for some, but not all, genes at 8C3/C4? The LCR encompasses more than 20 kb of DNA and is asymmetric with regard to nucleotide sequence and transcription factor binding sites. One explanation may therefore be that physical constraints intrinsic to the chromatin fiber allow one, but not the other LCR to correctly juxtapose itself relative to a given gene. Interestingly, the genes that respond to only a single LCR are always located downstream of that particular LCR. We found CTCF bound to the outer hypersensitive site 5 and we propose that CTCF-mediated loops may interfere with contacts between the LCR and some of the upstream genes.

How to interpret the looping out of 8C3/C4 from the CT particularly when it contains an integrated LCR? One possibility is that this reflects increased local decondensation of chromatin after the insertion of 5 additional (erythroid-specific) DNase I hypersensitive sites, which may result in increased mobility [17],[58]. Regulatory DNA elements, such as the LCR, serve as binding platforms for trans-acting factors that locally disrupt the nucleosome fiber, as revealed by DNaseI hypersensitivity, and cause decondensation of the region. Indeed, live cell imaging studies that measured the compaction of a transgene array demonstrated that decondensation was not dependent on transcription but was dictated by the binding of transcriptional activator proteins [59]. A region-wide increase in accessibility may facilitate the regulation of individual genes and the simultaneous increase in mobility of the locus may promote the interaction with RNAPII foci. The collective stabilization of a decondensed chromatin state could explain why housekeeping genes tend to cluster in the genome [15].

## Materials and Methods

### Gene targeting and the generation of transgenic mice

For the insertion of the human β-globin LCR into the mouse *Rad23a* gene, the *ClaI* Neo resistance cassette of an existing *Rad23a* targeting construct that removes *Rad23a* exon II–VII [36] was replaced by a *ClaI* fragment containing a PGK-Puro resistance cassette coupled to a 21.5 kb *SalI-ClaI* fragment containing the human β-globin LCR. Constructs with the *ClaI* fragment in opposite orientations were obtained: LCR-S, with hypersensitive site 1 of the LCR at the 3′-end of the *Rad23a* gene, and LCR-AS with HS1 of the LCR at the 5′-end of the *Rad23a* gene. Targeting in Ola129-derived ES cells, blastocyst injection to generate chimeric mice and breeding to obtain homozygous transgenic animals in an FVB background was done as described [36]. Genotyping was performed by Southern blot. Animal experiments were carried out according to institutional and national guidelines (Committee on Experiments with Laboratory Animals (DEC-Consult); Ministry of Agriculture, Nature and Food Quality, The Hague, The Netherlands).

### DNaseI hypersensitivity assays

DNaseI hypersensitivity assays were carried out on isolated nuclei from 6 E14.5 fetal livers. Nuclei were isolated in ice-cold lysis mix (10 mM Tris-HCl pH 7.5, 10 mM NaCl, 3 mM MgCl_2_, 0.1% NP40) by dounce homogenisation and subsequent slow spinning. Nuclei were incubated for 3 min at 37°C in lysis mix without NP40, substituted with 1 mM CaCl_2_ and increasing amounts of DNaseI. Reactions were stopped by adding equal amounts of 2× stop-mix (0.6 M NaCl, 20 mM Tris-HCl pH 8.0, 10 mM EDTA, 1% SDS), treated overnight with Proteinase K and DNA purified with phenol/chlorophorm. Samples were digested overnight (HS1, HS2: *PstI*; HS3, HS4, HS5: *HinDIII*), run on 0.7% agarose gels and visualized by Southern blotting. Primer sequences for Southern probes are presented in Table S1.

### Affymetrix gene expression analysis

Total RNA was isolated using the RNeasy Mini kit (Qiagen, Hilden, Germany) from livers and brains of three independent embryos and mice. Biotinylated cRNA was generated using the One-cycle Target Labeling and Control Reagents Kit (Affymetrix, Santa Clara, California, United States). All previous procedures and hybridization, washing and scanning of the Affymetrix Mouse Genome 430 2.0 Arrays were done according to manufacturers' instructions. Array-data was normalized using Bioconductor RMA ca-tools. For each probe set, the values of the three independent micro-arrays were averaged. When multiple probe sets represented the same gene, the highest value was chosen to represent the gene.

### Gene expression analysis

Total RNA was isolated as described previously (“Affymetrix gene expression analysis”). cDNA synthesis was performed using SuperScript II Reverse Transcriptase and Oligo(dT)_12–18_ primer according to the manufacturer's instructions (Invitrogen). Products were quantified by qPCR, using Platinum Taq DNA polymerase (Invitrogen) and SYBR Green (Sigma) on an Opticon 2 Real-Time PCR Detection System (BioRad). Primer sequences in Table S1. Transcript levels were normalized to the *Hprt1* transcript, encoding a relatively high expressed housekeeping gene on an unrelated chromosome, verified in the Affymetrix gene expression analysis not to be influenced by the integration of the human β-globin LCR (results not shown).

### Cell preparation and cryosectioning

For the preparation of cell blocks for cryosectioning, E14.5 fetal liver and brain tissues were fixed in 4 and then 8% paraformaldehyde in 250 mM HEPES pH 7.6 (10 min and 2 h respectively) [60]. Cell pellets were embedded in 2.1 M sucrose in phosphate-buffered saline (PBS) and frozen in liquid nitrogen as described previously [29]. Cryosections (140–180 nm in thickness, deduced from interference colour) were cut using an UltraCut UCT52 ultracryomicrotome (Leica, Milton Keynes, UK), captured in sucrose drops, and transferred to glass coverslips.

### Cryo-FISH

Cryo-FISH was performed as described previously [24]. A probe for the 8C3/C4 locus was obtained by labeling a BAC (RP24-319P23) with biotin or rhodamine by nick-translation (Roche). The BAC probe was co-precipitated with mouse Cot1 DNA (Roche; 1.7 µg/µl final concentration) and resuspended in either hybridization buffer (50% deionized formamide, 10% dextran sulfate, 2×SSC, 50 mM phosphate buffer pH 7.0) or a FITC-labeled mouse whole chromosome 8 paint (Applied Spectral Imaging, Israel). Probes were denatured at 70°C for 10 min, and re-annealed at 37°C for 30 min before hybridization. Probe specificity was confirmed on mouse spleen metaphase spreads.

### Immunolabeling

Immunolabeling of cryosections was performed as described previously [24]. The biotin-labeled BAC probe for the 8C3/C4 locus was detected using rhodamine-conjugated neutravidin (1/500; Molecular Probes), followed by a biotin-conjugated goat anti-avidin antibody (1/500; Vector) and rhodamine-conjugated neutravidin. Splicing speckles were detected with a human autoimmune serum against Sm antigen (1/2000; ANA-CDC), followed by a biotin-conjugated donkey anti-human antibody (1/100; Jackson ImmunoResearch, West Grove, PA, USA) and an AlexaFluor488-conjugated neutravidin (1/100; Molecular Probes). Serine 2 phosphorylated RNAP II was immunolabeled with H5 (1/1000; Covance, Berkeley, CA, USA), followed by an IgM-specific biotin-conjugated donkey anti-mouse antibody (1/250; Jackson ImmunoResearch) and Alexa 488-conjugated neutravidin or Alexa 647-conjugated streptavidin (1/100; Molecular Probes). After immunolabeling and before cryo-FISH, antibodies were fixed with 8% paraformaldehyde in 250 mM HEPES pH 7.6 (1 h), or with 2 mM EGS in PBS (30 min, 37°C).

### Microscopy and image processing

For confocal laser scanning microscopy, images were collected sequentially on a Leica TCS SP2 (100× PL APO 1.40 Oil objective) equipped with Argon (488 nm) and HeNe (543 nm; 633 nm) lasers. For wide-field light microscopy, images were collected sequentially on a Delta-Vision Spectris system (Applied Precision, Issaquah, USA) equipped with an Olympus IX70 widefield microscope (100× UPlanFl 1.3 Oil objective), a charge-coupled device camera, and the following filters: DAPI, FITC, RD-TR-PE, CY-5, CFP, YFP. The use of ultrathin cryosections allows for the use of wide-field microscopy with no reduction in axial (*z*) resolution and only a small reduction in lateral resolution. No bleedthrough was detected in these conditions, and images were collected without saturation of intensities. The images presented were contrast stretched, without further thresholding or filtering. For the generation of randomly positioned loci, the experimental images collected for the analysis of 8C3/C4 position relative to transcription factories were used. We developed a macro on ImageJ (Wayne Rasband, NIH, USA) that thresholds the DAPI signal and, for each locus, generates random coordinates until they fall within the mask of the corresponding thresholded nuclear section. These coordinates were used to measure the distance to the nearest transcription factory.

### Chromatin immunoprecipitation

ChIP was performed according to the Upstate protocol (http://www.upstate.com), with two modifications: (1) E14.5 fetal livers were made single cell by applying a cell-strainer cap (BD Falcon #352340, BD Biosciences, San Jose, California, United States) and (2) cells were fixed for 5 minutes in a 2% formaldehyde solution at room temperature. Chromatin fragments were quantified by qPCR (sequences of primers in Table S1) using Platinum Taq DNA polymerase (Invitrogen, Carlsbad, California, United States) and SYBR Green (Sigma, St. Louis, Missouri, United States) on an Opticon 2 Real-Time PCR Detection System (Biorad, Hercules, California, United States). Enrichments were calculated relative to the endogenous β-globin promoter and values were normalized to input measurements. Antibodies used: anti acetyl-Histone H3 (#06-599, Upstate, Charlottesville, Virginia, United States); anti K4 trimethyl H3 (#07-473, Upstate); anti C-terminal-Histone H3 (#ab1791; Abcam, Cambridge, United Kingdom); anti CTCF (H. Heath, manuscript in preparation).

### Chromosome Conformation Capture (3C) analysis

3C analysis was performed as described before, with slight modifications [51],[53],[54]. 3C material was digested using the restriction enzyme *BglII*. Ligation frequencies were quantified by qPCR (Opticon 2 Real-Time PCR Detection System, Biorad) using Platinum Taq DNA polymerase (Invitrogen) and double-dye oligonucleotides (5′FAM + 3′TAMRA) as probe (sequences of primers and probes in Table S1). To correct for differences in quality and quantity of templates, ligation frequencies between the fragments in the region on chromosome 8 were normalized to two fragments in the *Ercc3* locus, assumed to have a constant spatial organization independent of the presence of the human β-globin LCR. To correct for PCR amplification efficiency of different primer sets a *BglII* digested and re-ligated control template containing equimolar amounts of all possible ligation products was used. This control template was composed of two BAC clones containing all the analyzed fragments in the region (#RP24-136A15 and RP24-319P23, Ensemble Genome Browser), a construct containing the human β-globin LCR (see “Construction of targeting vectors”) and a PAC clone containing the *Ercc3* locus (#443-C18, MRC geneservice).

### Accession numbers

Micro-array data used for Figure 2: Gene Expression Omnibus (GEO) GSE5891 (E14.5 fetal liver and E14.5 fetal brain) and ArrayExpress E-MEXP-839 (adult liver) [61],[62].

ENSEMBL Gene Ids: *Ier2* (gene −7): ENSMUSG00000053560, *Btbd14b* (gene −6): ENSMUSG00000001910, *Trmt1* (gene −5): ENSMUSG00000001909, *Lyl1* (gene −4): ENSMUSG00000034041, *Nfix* (gene −3): ENSMUSG00000001911, *Dand5* (gene −2): ENSMUSG00000053226, *Gadd45gip1* (gene −1): ENSMUSG00000033751, (*Rad23a* (gene 0): ENSMUSG00000003813, *CalR* (gene +1): ENSMUSG00000003814, *Farsla* (gene +2): ENSMUSG00000003808, *Syce2* (gene +3): ENSMUSG00000003824, *Gcdh* (gene +4): ENSMUSG00000003809), *Klf1* (gene +5): ENSMUSG00000054191, *Dnase2a* (gene +6): ENSMUSG00000003812

## Supporting Information

Figure S1 Primary transcript levels of genes at 8C3/C4
(1.79 MB EPS)

Figure S2 Insertion of the LCR has no effect on the association of 8C3/C4 with splicing speckles
(0.81 MB EPS)

Table S1 Primers and probes
(0.06 MB DOC)
