# Correction: Transcription and Chromatin Organization of a Housekeeping Gene Cluster Containing an Integrated β-Globin Locus Control Region

**DOI:** 10.1371/annotation/b4241c5d-5403-4797-8579-913ef9be560b

**Published:** 2008-03-28

**Authors:** Daan Noordermeer, Miguel R. Branco, Erik Splinter, Petra Klous, Wilfred van IJcken, Sigrid Swagemakers, Manousos Koutsourakis, Peter van der Spek, Ana Pombo, Wouter de Laat

Ana Pombo (AP) should be added as one of the persons who wrote the paper.

