# Supplementary material for: Transcription and Chromatin Organization of a Housekeeping Gene Cluster Containing an Integrated β-Globin Locus Control Region
Source: PLoS Genet. 2008 Mar 7;4(3):e1000016. doi: 10.1371/journal.pgen.1000016 (PMC2265466; doi:10.1371/journal.pgen.1000016)
Supplement: Table S1 — Primers and probes (0.06 MB DOC) [file pgen.1000016.s003.doc]

**Supplementary table 1: Primers and probes**

# Gene expression analysis

| **Primer set** | Sequence |
| --- | --- |
| mRNA Ier2 | ATCACTACCGTCGCTCAA |
|  | TACGCAAGAGGAAGTGCT |
| mRNA Btbd14b | TTGCCACCCAAAGCTCTA |
|  | TTCATAAGCTGTGCCCTTG |
| mRNA Trmt1 | TATAACCCGGTGCAGGAAT |
|  | TTCTCTGAGTCCTTCTCTCC |
| mRNA Lyl1 | GACCCTTCAGCATCTTCC |
|  | CTGTTGGTGAACACTCGC |
| mRNA Nfix | GAACTGGACCTTTATCTGGC |
|  | CGCAACTGGAGTCTGTGA |
| mRNA Dand5 | CTTCTACATTCCCAGCTCG |
|  | CTGGACCAATACCGTGGA |
| mRNA Gadd45gip1 | AAAGCAGAAGCGAGAACG |
|  | ATAGCAGCAATTCGTGCC |
| mRNA CalR | GACTTTCTGCCACCCAAG |
|  | GTTCCCACTCTCCATCCA |
| mRNA Farsla | GAGTAGCCATGGCGGATA |
|  | CCAGCACTTGGTAGAACGA |
| mRNA Syce2 | TTGTACACCGTTTCCACAGT |
|  | GACCATGCACTTATGACCAA |
| mRNA Gcdh | CTGCCGATGAGAAACTGATA |
|  | TCGACCTGTAGCCACTGTC |
| mRNA Klf1 | CATCAGTACACTCACCACCCT |
|  | CGGAACCTGGAAAGTTTG |
| mRNA Dnase2a | TGCCAATCCTTGCAAACT |
|  | CGACCAACCTCCTAAATCC |
| mRNA Mast1 | TGGAAGGTGGTGACTGTG |
|  | AATTGTGTAAGTACTCAAGGGC |
| mRNA Hprt | AGCCTAAGATGAGCGCAAGT |
|  | ATGGCCACAGGACTAGAACA |
| Primary transcripts CalR | CATAGAATGGAGGACATCTGG |
|  | GTTCCCACTCTCCATCCA |
| Primary transcripts Nfix | CAGCCACATCACATTGGAG |
|  | CTGAACAAATACCAGCAACTG |
| Primary transcripts Rad23a | GGTGTCTTGGTGTGTTAGTG |
|  | GCTTCTATCTTCTCCTTCAGC |
| Primary transcripts Klf1 | CAGTGCCTACCATTCAAGC |
|  | AAGGGTCCTCCGATTTCAG |
| Primary transcripts Prdx2 | TTTCCTGTCTCTACCCGTG |
|  | ATAGAGGTCGTGATGAGGC |

# DnaseI hypersensitivity assay

| Primer set | Sequence |
| --- | --- |
| Human β-globin LCR HS1 | GAAGCCTCTGGTCAGCAT |
|  | CAGTGGTAGAAGCAGGAAGAT |
| Human β-globin LCR HS2 | GACACATGCTCACATACGG |
|  | AACCCTCACAGCTGCTAAC |
| Human β-globin LCR HS3 | AGGGCTCCAGCATGTAGA |
|  | TCAAGGTCGAAGGTAGGAAC |
| Human β-globin LCR HS4 | AATTTAAGCTCCAGTCTCTGC |
|  | TCCCATATTCTGAAGCATTC |
| Human β-globin LCR HS5 | CAGCCCTGAGCACTTACA |
|  | TTTCTCCTTTGTTGACCAAG |

# Chromosome Conformation Capture (3C)

| Primer | Sequence |
| --- | --- |
| 8C3/C4 fixed fragment | CCTTCCTCCACCATGATGA |
| 8C3/C4 fragment I | GCATATTTGACTTTTACAAGCTGG |
| 8C3/C4 fragment II | GTGGTAGCAGAAGTCTCAAG |
| 8C3/C4 fragment III | ACTCTTAAACTGGCTGTGATG |
| 8C3/C4 fragment IV | CGGGTCACGAGATTCAGA |
| 8C3/C4 fragment V | AGTTCCTAGCCGTTCCTTAG |
| 8C3/C4 fragment VI | CAGAATGGGTCTATGGAAAGAG |
| Ercc3 fragment I | ATGGCCTGAAGAAACCGC |
| Ercc3 fragment II | CTTAGGCAACACACTCAAGC |
| Probe | Sequence |
| 8C3/C4 double-dye probe | AAAGCTTAGGGCTCCAGCTTCCC |
| Ercc3 double-dye probe | CCATCTTCCACATCAATGGGACCC |

# Chromatin immunoprecipitation

| Primer set | Sequence |
| --- | --- |
| Promoter Lyl1 | TTCAAAGCTAGACCAACCTCA |
|  | CACAGCACTGGAAGACCC |
| Promoter Nfix | TCCAAACCACACTTCAGTAG |
|  | GAAGGAGAAACACAGCGTT |
| Intergenic region Nfix - Dand5 | ACAGAGACAGCCGAATACC |
|  | GACCTCCCTCTGTCTGTTC |
| Promoter Dand5 | AACTCTCAAGCTGCTCTCC |
|  | CTGTGCAGTCGTTTGTCTG |
| Promoter Gadd45gip1 | TCGGAGGGTAAAGGCATT |
|  | AGTGTTGAAGTGTTGGTGAT |
| Promoter Rad23a | CACCAAGACAGCGGAATG |
|  | GGCTGCACCTTACCTTAGA |
| Promoter CalR | CTGGGAAGCAATGGAAAG |
|  | TTATATTCACCTACCTCTCACCC |
| Intergenic region CalR - Farsla | AGCACCTTCTGACTTCCAA |
|  | GGAGAGATGGCTGAGAGTAA |
| Promoter Farsla | AACTAAAAGCCACTGGGGT |
|  | TAAGTGTGGCAAATGAGCC |
| Promoter Syce2 | CGCACCTACGCATTATGA |
|  | TGCCTTTTGGGCTATGCT |
| 3' Syce2, 3' Gcdh | ATTGCTCTCCCAAGGATCA |
|  | TGAGCTGAAGATTCCAAACC |
| Promoter Gcdh | GGAACCATAACCTGGAAGGG |
|  | AAGGAGGAACCAATGAGCAA |
| Promoter Klf1 | CTTTGCCTGGGTCTTATCA |
|  | TCCTCTCTCTCTTCTGAATC |
| Promoter Dnase2a | GGGTCACGAGATTCAGATG |
|  | GCTTCGTCTCCACCCTCG |
| Intergenic region Mast1 - Rtbdn | ATGCTCAGCCAGTAGTAGTT |
|  | TTTACTCTGTGGGTCCTGG |
| Promoter Prdx2 | ATGCCCGGATTCCAACCG |
|  | TCCACACGCTTTCACAAG |
| Human HS5 | CTGAAGCTGCTGTTATGACC |
|  | ACAACTTCCTTGCTTGGG |
| Mouse HS5 | TTCACCACTAGAGGGAAGG |
|  | GAACGTGCAAGTGTCCTG |
| Promoter Amylase | CTCCTTGTACGGGTTGGT |
|  | AATGATGTGCACAGCTGAA |
| Promoter Beta Major | GGGAGAAATATGCTTGTCATC |
|  | CAACTGATCCTACCTCACCTT |
